# Stress-Related Behaviors in Companion Dogs Exposed to Common Household Noises, and Owners' Interpretations of Their Dogs' Behaviors

**DOI:** 10.3389/fvets.2021.760845

**Published:** 2021-11-08

**Authors:** Emma K. Grigg, Juliann Chou, Emily Parker, Anwyn Gatesy-Davis, Sara T. Clarkson, Lynette A. Hart

**Affiliations:** ^1^Department of Population Health and Reproduction, School of Veterinary Medicine, University of California, Davis, Davis, CA, United States; ^2^Animal Biology, College of Agricultural and Environmental Sciences, University of California, Davis, Davis, CA, United States; ^3^Department of Animal Science, University of California, Davis, Davis, CA, United States

**Keywords:** companion animal behavior, dog, noise sensitivity, welfare, *Canis familiars*

## Abstract

Sudden, loud noises are one of the most common triggers for fearful behaviors in dogs, and many companion dogs suffer from noise sensitivity. Existing research focuses on dramatic infrequent sounds (e.g., thunderstorms, fireworks). Anecdotally, and based on reports of undesirable behaviors in response to noises in the home, many common household noises may also be causing fear and anxiety in companion dogs. However, these responses have not yet been studied in home environments. We surveyed 386 dog owners about their dogs' responses to household sounds, and recorded dog behaviors and human reactions from 62 videos and compilations available on an online video sharing platform, featuring dogs reacting to common household noises. Numerous signs of canine fear and anxiety were reported by survey respondents and observed in the videos, in response to both daily, and irregular but “normal,” household noises. Responses were significantly stronger to sounds characterized as high frequency intermittent than to sounds characterized as low frequency continuous. Respondents appeared to underestimate their dogs' fearfulness, and the majority of humans in the videos responded to their dogs' behaviors with amusement; welfare concerns were rarely expressed. While these videos cannot be used to calculate actual prevalence of these issues, our data support that some owners are underestimating fearfulness in their dogs in response to household noises, and responding inappropriately to dogs' expressions of fear and anxiety. Better education is required for dog owners to accurately interpret canine body language, to both safeguard dogs' welfare and minimize development of anxiety-related behavior problems.

## Introduction

The fear response is a normal, adaptive behavior that helps protect the individual from harm; however, fear responses can be considered abnormal when they are repeatedly and consistently triggered by non-threatening stimuli, or when the level or duration of response is out of proportion compared to the actual threat level posed by the stimuli (1, 2). Sudden, loud noises are one of the most common triggers for fearful behavior in dogs (1, 3), and considerable research supports the concept that many companion dogs suffer from noise sensitivity and are affected by salient noises. Reported prevalence of noise sensitivities (marked and even extreme responses to noise) in domestic dogs varies by study, but often ranges as high as 50% (4–7).

Loud, sudden (and often unexpected) sounds have been used in studies of behavioral and physiological stress responses in dogs (8, 9). That research focuses on dramatic infrequent sounds—sounds that dogs do not encounter daily or on a regular basis, but rather occur as sporadic, unpredictable events [e.g., thunderstorms (8, 10, 11), fireworks (12, 13), and gunshots (9)]. Canine reactions associated with these stressors have been interpreted by professionals as expressions of stress, anxiety, and fear (11). For example, physiological responses to loud noises can include a dramatic (207%) increase in salivary cortisol, lasting for 40 mins or more (8); unexpected noises resulted in rapid responses including tachycardia, hypertension, and increased secretion of epinephrine and norepinephrine (14); and exposure to acute, irregular noises is associated with stimulation of the HPA axis and a rapid (within 15-min) increase in circulating cortisol concentrations (15, 16). Behavioral responses to loud noises include panting, hiding, pacing, cowering/lowered body posture, shaking/trembling, barking, escape attempts/retreating, and seeking out familiar people (2, 5, 8, 17).

Stress associated with fear and anxiety can have negative impacts on health, welfare, behavior and lifespan (18, 19), depending on both the nature of the stressor (intensity, duration, persistence, etc.) and the coping skills of the individual (20, 21). Every day, companion dogs are exposed to common household noises. Anecdotally [e.g., (22)], and based on reports of undesirable reactions to noises in the home [e.g., (23)], many of these noises may be causing fear and anxiety in the resident dogs. In this study, we define “common” household sounds as including both those that occur daily, and also other common, but non-daily, sounds that are heard in the home and confined to the space where a pet resides. The response of companion dogs to common household noises has not yet been studied in the dogs' home environments. Dogs' reactions to these household noises could have implications for their health and wellbeing in human homes. If these dogs are experiencing fear in response to regularly-occurring household stimuli, they may as a result be experiencing reduced welfare, and be at risk for the development of stress-related behavioral or physiological problems (24). Damage to the human-animal bond which can result from undesirable behaviors associated with fears and phobias can lead to decreased commitment to care of the dog, and/or an increased risk of relinquishment or euthanasia (19).

The purpose of this study was to investigate and describe, using survey data, whether household dogs display a fear/anxiety/stress response to common sounds occurring within homes. In addition, we recorded both canine and human behavior in publicly-shared videos and video clip compilations which featured companion dogs reacting strongly to household noises. Our goal was to describe both the prevalence and nature of the dogs' reactions to household noises seen in these videos, as well as the various interpretations that owners offered in response to their dogs' displays, possibly indicating fear, anxiety, or stress. Understanding the significant or insignificant effects of household sounds/noises on a companion dog's behavior and mental state will allow for better understanding of dogs' behaviors and how to improve their general welfare at home.

Our hypotheses were that: (1) Sounds that are common in households can elicit fear, anxiety, or stress responses in companion dogs; and (2) Owners can misinterpret or respond negatively to expressions of fear, anxiety, or stress in their companion dog if the stressor is considered “common.”

## Materials and Methods

### Owner Surveys

An online, anonymous, cross-sectional survey was developed using Qualtrics (Qualtrics, Inc., Provo, UT, USA). This short survey was designed, reviewed, and tested by the authors (EP, LAH, AG-D), and was pilot tested by 12 local dog owners for ambiguity and/or potentially missing or inappropriate response options, with revisions made in an iterative process based on the results of this pilot testing and initial viewings of the online videos (see below). The study design and the final 23-item survey (see Supplementary Material 1) were approved by the University of California, Davis, Institutional Review Board (IRB # 1469462-1). Survey respondents were recruited *via* social media (primarily Facebook; Menlo Park, CA) in the Fall of 2019. No mention of noise or noise phobias was made in the initial recruitment materials; the survey goal was described on the consent form as exploring how pet dogs in the home respond to various sounds.

Descriptive statistics were then calculated on dog demographics, frequencies and types of behavior reported by owners (and the sound sources to cause reactions), strength of the dog's reactions, and owner attitudes toward their dog's behaviors. We also assessed whether strength of the dogs' reactions was associated with the nature of the sound stimulus, using Chi-square analyses.

### Online Videos

In addition, we used an online search engine (Google.com; Mountain View, CA) to search for individual videos and video clip compilations posted to an online video sharing and social media platform (YouTube.com; San Bruno, CA) featuring dogs reacting to household sounds. Search phrases (including word substitutions used) included: “Dog afraid (or ‘scared’) of sound (or ‘fire alarm,’ ‘beeping noise,’ ‘smoke detector,’ ‘smoke alarm,’ ‘vacuum’),” “Dog fear household sound,” “Dog beep alarm fear,” “Dog fire alarm,” “Why is my dog scared (or ‘afraid’) of the smoke (or ‘fire’) alarm,” “Dog afraid of beeping noise,” “Dog and vacuum,” “Dog hates fire alarm (or ‘vacuum,’ ‘smoke detector,’ ‘beep’).”

#### Individual Videos

Behavioral data from all videos was then coded, using an ethogram of 24 behavioral signs of stress in dogs (Table 1A), including both overt and more subtle fear-related behaviors and postures [e.g., (24)]. Frequency of behavioral “events” of short duration (e.g., lip lick, jumping in place), and duration of behavioral “states” (e.g., panting, barking, maintaining proximity to owner), were recorded using an all-occurrence approach by two trained observers, after testing for inter-rater reliability using Pearson's correlation coefficients (25). In order to allow comparison of dogs' behavior between video clips of varying duration, behavioral data were converted to rates per minute of usable video footage: frequency of behavior/min (for behavioral events) or duration (sec) of behavior/min (for behavioral states). Times when the dog was not clearly visible in the video (i.e., out of view) were removed from the analysis. For behaviors requiring a clear view of one specific part of the dog (e.g., face, tail), times when this part of the dog was out of view were also removed from analysis for that behavior (e.g., times when the tail could not be seen were removed from frequency/duration calculations for tail tuck, tail wag). The type of sound stimulus in the video (e.g., smoke detector chirp/low battery indicator, fire alarm, vacuum cleaner) was noted, as well as the primary characteristics of the sound audible in the video (e.g., high frequency, low frequency, intermittent, continuous; Table 2). Frequency of the sounds in the videos was categorized according to perceived pitch; given quality variations of sound in the videos, we did not attempt to measure exact frequencies or levels of the sounds audible in the videos. General reactions of the humans featured in the videos (e.g., concern, amusement; Table 1B) were also noted, using a one-zero approach.

**Table 1:** Canine (A) and human (B) ethograms used in recording behavior from the videos and video clip compilations.

	**Operational definition**
**(A) CANINE BEHAVIOR**	
**Vocalization**	
Barking	Clear, short and repetitive vocalizations directed toward stimulus
Howling	Prolonged high-amplitude vocalization, snout held high, lips drawn together while exhaling, mouth open
Whining	A cyclic low-amplitude vocalization, mouth closed
**Body movement**	
Pacing	Repeated movement from one position to another two or more times in a fixed route, includes circling the span of the room
Retreating	Accelerated movement away from the stimulus and remains distant from general proximity of the stimulus
Hiding	State of concealing physical body from the stimulus
Trembling/Shaking	Visible vibrations of the entire body, excluding the tail (when visible, verbalized or noted by owner in video)
Spinning	Turning or whirling quickly around a center of axis, similarly to a dog chasing its tail
Digging	Forelimb paws alternating repetitively, making contact with source of stimulus
Jumping on owner	Standing on hind legs with forelimb paws risen off the ground and makes contact with the owner
Jumping in place	Jumping intentionally into the air, landing on the ground in the same approximate area (either all 4 or 2 paws off the ground)
Jumping (Misc.)	Jumping intentionally into the air, landing on furniture/objects OR further than 1 meter from start point (either all 4 or 2 paws off the ground)
Lunging	Attempt to bite while moving in the direction of stimulus without physical contact
Proximity	In contact with or within 1 body length of the owner
Frozen	General rigidity of body posture with fixed attention in the direction of the stimulus (>3 s)
**Facial orientation**	
Lip licking	Showing one or more brief movements of the tongue toward the nose or corners of the mouth
Panting	Tongue exposed with audible and/or observable heavy breathing
Salivating	Mouth tense and pulled back, presence of excess saliva around the mouth
Tucked ears	Ears folded against sides and/or back of head and having a flattened appearance, excluding tucked ears when howling
Yawning	Opens mouth widely and inhales
Fear grimace	Corners of the mouth pulled back
Agonistic pucker	Corners of the mouth pushed forward
**Tail orientation**	
Tail Wag	Regular movement of the tail from side to side, relaxed or tense
Tucked tail	No movement of tail tightly between hind legs, may be curled under ventral side
**(B) HUMAN REACTION**	
Concern	Vocalization indicating concern for the welfare of the subject
Amusement	Cheery vocalization indicating laughter or chuckling as a reaction to the subject
Analysis	Commentary regarding the description of subject behavior, posture, and overall reaction to stimulus
Antagonist	Deliberately manipulating or instigating stimulus in order to induce response from subject
Preventative inhibitor	Interference in order to reduce or stop subject behavior toward stimulus (removing stimulus, removing dog, physically separating, etc.)
Spectator	Person is present with no vocalization or interference with subject

**Table 2:** Primary characteristics of the household sounds audible in the videos and video clip compilations.

**Sound characteristics**
High frequency
Low frequency
Unknown frequency
Continuous
Repetitive/Intermittent
Unknown duration
Sudden/Loud Noise
Human Noise^a^

a *Human noise includes (loud) voices, furniture being moved, crowds, people moving around in other rooms, voices of visitors*.

Descriptive statistics were calculated on distribution and relative frequencies of sound sources, observed canine stress behaviors, and human reactions in these videos. In addition, frequency/duration of occurrence of all recorded canine stress behaviors was compared, using Mann-Whitney U non-parametric t-tests, between the two most common sound source categories.

#### Video Clip Compilations

Video clip compilations consisted of collections of much shorter clips featuring only the most extreme dog reactions to household sounds; these clips had potentially been excerpted from longer videos by the individuals assembling the compilations. Both behaviors of dogs and reactions of the humans (Table 1) in the video clip compilations were coded using a one/zero approach. As with the video clips, the type of sound stimulus in the video was noted, as well as the primary characteristics of the sound audible in the video (Table 2). Descriptive statistics were calculated on distribution and relative frequencies of sound sources, observed canine stress behaviors, and human reactions in the video clip compilations.

All statistical analyses were conducted in XLSTAT 2021 (Addinsoft, Inc, New York, NY, USA) for Microsoft Excel (Microsoft Corp., Redmond, WA, USA), with α = 0.05.

## Results

### Owner Surveys

Survey responses were obtained from 386 current dog owners, reporting on dogs of 72 different breeds. The majority of dogs were of mixed breed (*n* = 191, 50%); percentage of each breed fell sharply after that (e.g., Labrador retriever *n* = 22, 5.8%; American pit bull terrier, Golden retriever, and Australian shepherd tied at *n* = 11, 2.9%). Most dogs were medium-sized (mode = 40–60 lbs. or 18–27 kg, *n* = 110, 28.6%), adult (mode = 2–7 years, *n* = 163, 42.6%), and the only dog in the household (*n* = 228, 59.2%). Just over half the dogs were obtained from a shelter or rescue (*n* = 195, 50.5%).

Owner reports of fear-related behaviors in their dogs were mixed; most owners reported that their dogs were not fearful (*n* = 255, 66.8%), and correspondingly, most had not sought help for a behavioral issue (*n* = 269, 70.0%). However, when asked if they consider their pet to have a fear of loud noises (such as fireworks, thunderstorms, gunshots), responses were equally divided between yes and no (both *n* = 170, 47.5%). When asked which (if any) category of household sounds their dog reacted to, and which (if any) noises evoked an extreme response in their dogs, reactions to “loud/rare noises” were the most commonly reported (*n* = 199, 51.6%), with 7.3% (*n* = 28) of all respondents reporting extreme reactions to these types of household noises (Table 3). The strength of the dogs' reactions was significantly associated with the nature of the sound stimulus (χ^2^ = 7.972, df = 2, *p* = 0.019), with extreme reactions significantly more likely with sound sources characterized as “beeping” (smoke detectors, etc.) (Table 3). Note that human noise/door knocking was not included in the chi-square analysis, because reactions were only reported when the dog's reaction was intense (and many respondents indicated that they considered barking at the door to be a typical dog behavior).

**Table 3:** Frequencies of dogs' reactions and extreme reactions to common sounds reported by dog owners (*n* = 386) (contingency table).

	**Household sound category**				
	**Loud/rare sounds (e.g., fireworks, thunderstorm, gunshots)**	**Beeping sounds (usually higher frequency: e.g., alarm clocks, smoke detectors, cell phone noises)**	**Appliance sounds (usually lower frequency: e.g., laundry washing machine/dryer, dishwasher, vacuum cleaner, fans, plumbing)**	**Human noise^a^^,^^b^**	**Door knock^a^**
Owners reporting dogs react to sound	199	112	69	10	5
Owners reporting extreme reaction to sound	28	34	12	10	5

*Extreme reactions were significantly more likely with sound sources characterized as “beeping” (χ^2^ = 7.972, df = 2, p = 0.019)*.

a *Common sound stimulus examples reported in the “other: describe” text box*.

b *Human noise includes (loud) voices, furniture being moved, crowds, people moving around in other rooms, voices of visitors*.

The most commonly reported reaction to household sounds was barking (*n* = 193 or 50.0% of owners reported, 29.0% of all reactions), followed by retreating (*n* = 87 or 22.5% of owners reported, 13.1% of all reactions) and pacing (*n* = 63 or 16.3% of owners reporting, 9.5% of all reactions) (Table 4). Indicators of marked stress in dogs (shaking/trembling, hiding, howling, etc.) were also reported, but by smaller numbers of dog owners (Table 4).

**Table 4:** Types and frequencies of stress-related dog behaviors reported by owners (*n* = 386) in response to household noises.

	**Frequency**	**Percentage of total behavioral reactions reported (*n* = 665)**	**Percentage of participants (*n* = 386) reporting this reaction**
Barking	193	29.0	50.0
Retreating	87	13.1	22.6
Pacing	63	9.5	16.3
Whining	56	8.4	14.5
Hiding	56	8.4	14.5
Tucked ears	51	7.7	13.2
Shaking	50	7.5	13.0
Panting	34	5.1	8.8
Yawning	26	3.9	6.7
Lip lick	22	3.3	5.7
Howling	17	2.6	4.4
Salivate	10	1.5	2.6

### Online Videos

#### Individual Videos

Behaviors from a total of 57 videos were recorded (see Supplementary Material 2), with a total duration of 64.4 mins; after times when the dogs were out of view were removed from analysis, 50.3 mins of usable video data remained. Inter-rater reliability (*r*) was 0.97 (*p* < 0.0001, *n* = 25) for behavioral states, and 0.75 (*p* < 0.0001, *n* = 45) for behavioral events. Sound sources causing stress-related dog behaviors in these videos are shown in Table 5. In 82.5% of the videos, the sound was categorized as high frequency intermittent (HFI)—e.g., smoke detector warning beeps or “chirps”; 10.5% were categorized as low frequency constant (LFC)—e.g., microwave humming, robot vacuum cleaner. In 7.0% (*n* = 4) of the videos, the sound could not be heard in the video and thus the primary sound characteristics could not be reliably determined by the video coders. These videos were removed from the analysis for the Mann-Whitney U comparisons involving sound source.

**Table 5:** Descriptions of sound sources^a^ causing stress-related dog reactions in the YouTube videos (*n* = 57).

**Sound source**	**Frequency**	**Relative**
		**frequency (%)**
Smoke alarm	29	50.9
Smoke detector chirp	13	22.8
Smoke detector alarm with voice	5	8.8
Microwave humming	3	5.3
Automated vacuum cleaner (e.g., Roomba™)	1	1.8
Carbon monoxide test beep	1	1.8
Microwave beeping	1	1.8
Sirens	1	1.8
Smoke alarm siren	1	1.8
Smoke detector (no sound audible; response was to owner approaching device)	1	1.8
Vacuum cleaner	1	1.8

a *Sound source information was obtained from audible sound within video itself (93% of videos), verbal description within the video or video title (4% of videos)*.

The most frequently recorded behavioral states in the videos were proximity to owner (defined as the dog maintaining a distance of ≤1 body length from the owner), tail wagging, howling, barking and panting (Table 6). The most commonly recorded behavioral events were lip licks and jumping in place. “Other” behaviors not originally included in the ethogram, but seen occasionally in the videos, included growling, paw lifts, and lowering of head or body.

**Table 6:** Behaviors recorded in the YouTube videos (*n* = 57), in order of frequency.

**Dog behavior**	**Totals**	**Mean/min**	***Std dev***	***n* (#videos behavior seen)**	**Percentages (%videos behavior seen)**
***Proximity to owner***	1388.0	24.4	23.9	40	70.2
***Tail Wagging^a^***	913.7	16.0	21.9	25	43.9
***Howling***	475.9	8.3	12.7	24	42.1
***Barking***	471.0	8.3	11.7	28	49.1
***Panting***	351.1	6.2	18.9	9	15.8
***Whining***	326.8	5.7	15.8	19	33.3
***Tucked Ears***	272.1	4.8	12.9	12	21.1
***Other state^b^***	184.1	3.2	9.8	11	19.3
**Lip Lick**	140.4	2.5	4.3	25	43.9
**Jump in place**	125.1	2.2	8.3	8	14.0
***Digging***	87.2	1.5	6.9	5	8.8
***Trembling***	60.0	1.1	7.9	1	1.8
***Retreating***	59.0	1.0	4.4	5	8.8
***Pacing***	43.9	0.8	3.4	4	7.0
***Frozen***	35.1	0.6	4.4	2	3.5
**Jump (misc)**	34.8	0.6	1.9	10	17.5
***Spinning***	32.6	0.6	3.7	3	5.3
**Other event^c^**	24.3	0.4	2.3	4	7.0
**Lunge**	19.1	0.3	2.5	1	1.8
**Fear grimace**	14.3	0.3	1.2	3	5.3
**Jump on owner**	6.4	0.1	0.5	3	5.3
**Yawn**	4.0	0.1	0.3	3	5.3
***Hiding***	2.9	0.1	0.4	1	1.8

*All behaviors were standardized to rate/min (frequency/min for event behaviors; duration/min for state behaviors, in italics) that the dogs were visible in the videos*.

a *Given low quality of many videos, valence of tail wags (tense vs. relaxed) could not be reliably coded*.

b *Growling, air licking, lowered head/body posture*.

c *Paw lift, head tilt, head shake*.

For the comparison of behaviors in response to the two most common categories of sound (HFI vs. LFC), the behaviors hiding (not seen in the videos featuring HFI or LFC sound sources), frozen (only seen in one video), and other (because this category contained more than one behavior type, each of which was relatively rare) were removed from the analysis. Occurrence of only four of the dog behaviors differed significantly (at *p* < 0.0001) by sound category (Table 7): trembling (higher in response to HFI sounds); and retreating, lunging, and jumping on the owner (higher in response to LFC sounds).

**Table 7:** Results of the Mann-Whitney U test comparisons between rates of behavior (per minute) by sound source category, HFI (high frequency intermittent) vs. LFC (low frequency continuous); *n* = 54 videos.

**Behavior**	**HFI (mean ± sd)**	**LFC (mean ± sd)**	**HFI - LFC**	**Higher for**
**Jumping on owner^a^**	**0.1 ± 0.5**	**0.3 ± 0.7**	**<0.0001**	**LFC**
Jumping in place	2.6 ± 9.1	0.3 ± 0.7	0.922	
Jumping (misc)	0.7 ± 2.1	0.0 ± 0.0	0.615	
**Lunging^b^**	**0.0 ± 0.0**	**3.2 ± 7.8**	**<0.0001**	**LFC**
Lip licking	2.1 ± 3.2	1.7 ± 2.7	0.752	
Yawning	0.1 ± 0.3	0.0 ± 0.0	0.616	
Fear grimace	0.3 ± 1.3	0.0 ± 0.0	0.616	
Barking	8.1 ± 12.2	9.2 ± 2.7	0.161	
Howling	9.6 ± 13.2	4.1 ± 10.1	0.246	
Whining	6.9 ± 17.2	0.0 ± 0.0	0.141	
Pacing	0.9 ± 3.8	0.0 ± 0.0	0.782	
**Retreat^c^**	**0.7 ± 4.2**	**4.4 ± 6.3**	**<0.0001**	**LFC**
**Trembling^d^**	**1.3 ± 8.8**	**0.0 ± 0.0**	**<0.0001**	**HFI**
Spinning	0.7 ± 4.1	0.0 ± 0.0	0.616	
Digging	1.9 ± 7.5	0.0 ± 0.0	0.931	
Proximity	24.0 ± 23.8	21.1 ± 23.2	0.764	
Panting	5.8 ± 17.8	0.0 ± 0.0	0.710	
Tucked ears	4.2 ± 12.0	0.9 ± 2.1	0.783	
Tail wag	16.5 ± 23.1	15.1 ± 16.7	0.977	

*Significant differences are shown in bold*.

a *(U = 123.5, p < 0.0001)*.

b *(U = 117.5, p < 0.0001)*.

c *(U = 77.5, p < 0.0001)*.

d *(U = 144.0, p < 0.0001)*.

Human (owner) reactions to the dogs' behaviors in the videos varied, and more than one type of reaction was often observed within a single video (e.g., concern, amusement and analysis; or amusement and antagonist). The most commonly-observed reaction was “spectator” (the person is present with no vocalization or interference with subject), seen in 49.1% of videos; perhaps not surprising as the dogs were being deliberately filmed by owners presumably for sharing the dog's behavior online. The second most common reaction was amusement, seen in 45.6% of videos. In 26.3% of videos, the human narrator attempted to analyze or understand the dog's behavior; in 22.8% of videos, the human deliberately antagonized the dog to get the desired reaction. Concern for the dog was only expressed in 17.5% of the videos.

#### Video Clip Compilations

Five video clip compilations posted on YouTube were also coded; total duration of all compilations was 28.1 min. These compilations incorporated a total of 79 shorter video clips ranging in length from 3 s to 1.28 min. In all the compilation videos, sound was categorized as low frequency constant (LFC), and were almost exclusively vacuum cleaners: 48.1% of videos featured dog reactions to a robot vacuum cleaner (e.g., Roomba™), and 30.4% of videos featured an upright vacuum cleaner, with an additional 24.1% featuring just the nozzle of a vacuum cleaner (often used to interact with/antagonize the reacting dog). The remaining sound sources in the compilations were vacuum cleaners of unknown type (not visible in the compilation clips; 2.5%), and leaf blowers (1.3% of video compilation clips).

Dog stress-related behaviors recorded in the video clip compilations, in order of frequency, are shown in Table 8; behaviors not seen in any of the clips included jumping on owner, howling, pacing, fear grimace, trembling, or salivating. The most common behaviors seen in the compilations were lunging (18.3% of all behaviors) and barking (16.3% of behaviors) (Table 8). Although barking was also commonly seen in the individual videos (seen in 49.1% of videos; Table 6), lunging was very rare in the individual videos (1.8% of videos).

**Table 8:** Stress-related dog behaviors (*n* = 257) recorded in the YouTube video compilation clips.

**Behavior**	**Frequency**	**Percentage (of behaviors)**
Lunging	47	18.3
Barking	42	16.3
Tail wag	35	13.6
Proximity	26	10.1
Biting (vacuum)	18	7.0
Lip licking	13	5.1
Retreating	13	5.1
Jumping in place	12	4.7
Tucked ears	12	4.7
Jumping (misc.)	8	3.1
Agonistic pucker	6	2.3
Whining	6	2.3
Panting	5	1.9
Growling	5	1.9
Hiding	2	0.8
Spinning	2	0.8
Digging	2	0.8
Yawning	1	0.4
Frozen	1	0.4
Tucked tail	1	0.4

A total of 94 reactions by humans in the video clip compilations were recorded. As with the individually-posted videos (summarized above), spectator was the most common human reaction recorded (in 47.9% of compilation clips), followed by amusement (21.3% of compilation clips). The dog was deliberately antagonized to get the desired reaction in 20.2% of the compilation clips, and the human attempted to analyze the dog's behavior in 7.4% of the compilation clips. Concern for the dog was not expressed/observed in any of the compilation clips.

## Discussion

Publicly-shared videos do not represent systematic or random sampling, and so cannot be used to estimate the true prevalence of fear in response to common household noises in companion dogs. Owners of dogs who exhibit more extreme reactions to common household items that the owners consider “normal” are presumably far more likely to film and share their dog's behavior online. Nonetheless, numerous canine behaviors frequently associated with fear and anxiety (both overt and subtle) were observed in these videos, in response to both regular (e.g., microwaves, vacuum cleaners) and irregular but “normal” household noises (e.g., smoke detector low-battery warning beeps or chirps, smoke alarms). Dog behaviors in response to these noises included proximity-seeking directed toward their owners, an attachment-related behavior seen in dogs (and humans) as a means of coping with present stress (26–28), as well as other recognized signs of canine fear and anxiety, such as panting, howling, and lip licking. Behavioral signs of fear and anxiety in response to household noises were also reported by dog owners in the survey data, particularly when the sound was loud/infrequent (Tables 3, 4), although our brief survey did not enable detailed analyses of differences between the types of behavioral reactions associated with specific sound sources. While some of the behaviors recorded in the videos (e.g., barking, lunging, jumping on owner) can reflect other canine emotions such as excitement, many of the behaviors seen (e.g., trembling, panting, retreating from the sound source) are commonly associated with noise phobias (29) and are likely to reflect distress in these videos, given the context in which the behaviors were seen. Our Hypothesis 1 was supported by our data: certain types of household noises do cause marked fear in some dogs, and future research into the true prevalence of this issue, and the associated welfare issues, is warranted.

Some categories of household noises appear to be more problematic for the dogs. More intense signs of fear, such as trembling, were significantly more likely to be seen in the presence of sounds characterized as high frequency intermittent (HFI), such as smoke detector beeps, in the videos; and, in the owner surveys, extreme reactions were significantly more likely to be reported by dog owners in response to these types of sounds. Behaviors associated with arousal, agitation, and excitement (barking, lunging) were more commonly seen in the presence of low frequency constant (LFC) sound sources, such as vacuum cleaners, although behavioral signs of fear (such as lip licking, ears tucked back) were also seen with LFC sounds. This pattern is also seen in the differences in dogs' behavior in the compilations (which all featured LFC sound sources, primarily vacuum cleaners) vs. the individual videos (the majority of which featured HFI sound sources). For example, lunging was the most commonly observed behavior in the video compilation clips (Table 8), but was very rare in the individual videos (Table 6). Dogs' reactions to vacuum cleaners in these videos, exhibited in behaviors such as lunging and barking, may stem from emotional reactions other than fear, as has been noted elsewhere (7). Videos consistently presented the more extremely stressed dogs responding to the higher frequency beeps occurring sporadically, whereas the compilations depicted dogs' less-extreme responses to lower frequency appliance sounds that occur on a more regular basis.

The more extreme reactions to HFI sounds in this study are in agreement with the literature on noise sensitivity in dogs: loud noises, especially when unpredictable and when the dog concerned cannot control their exposure to the sound (as is the case here, for dogs confined inside a human dwelling), can cause intense fear reactions in dogs, detectable in both physiological and behavioral measures (15, 24, 30). The frequency, and constancy, of the sounds is an important factor in the dog's reaction. High frequency sounds are believed to be more salient for many species (i.e., attracting greater attention and prompting greater alertness) than lower frequency sounds (31); for example, the cries of a human baby will cause distress to a dog, despite the fact that human infant vocalizations are not of direct evolutionary importance to dogs (32, 33). Dogs' sensitivity to high frequency sounds is greater than humans', whereas at lower frequencies, the sensitivities of dogs vs. humans may not differ much (31, 34). In addition, the amplification processes of the ear may be stronger in dogs than humans (31), thus dogs could be more adversely affected by high frequency sounds in the home than their human companions.

Smoke detectors and smoke alarms, while an important safety measure in the home, are a particular concern for companion dogs. These devices are designed to effectively wake up people from deep sleep, and are thus commonly set at a frequency of around 3,000 Hz, designed to achieve a sound level of at least 75 dBA at a person's pillow (necessarily being much higher at the source) (35). Dogs, regardless of their body size, have particularly high sensitivity for hearing in the range 1,000–8,000 Hz (36), and dogs generally have better hearing in these high ranges than humans (31). Further, a significant proportion of adult humans over 48 years of age experience some degree of hearing loss (37): most likely involving sensitivity to higher frequencies. Thus, smoke alarms that are somewhat loud to most humans are likely to be painfully loud to most dogs.

This influence of the nature (frequency, constancy, predictability) of the sound source may also explain differences between our study and Gin et al. (16), who found that companion dogs did not have a significant stress response to vacuum cleaner sounds in a veterinary hospital setting, based on serum cortisol levels collected after exposure to the sounds. Gin et al. (16) noted a number of possible explanations for the lack of anticipated increases in cortisol levels, including lack of pre-exposure cortisol measurements, familiarity of these particular dogs with the clinic setting, and presence of owners as a mitigating influence on the dogs' stress levels. In addition, however, it may be that the characteristics of the vacuum used in that study may not have been the characteristics which cause the strongest fear reactions in dogs. Similarly, Stellato et al. (38) found that only respiratory rate increased when dogs were exposed to background noises (people talking, dogs barking, metal doors banging) during veterinary exams in a clinic setting. Behavior and other physiological variables (e.g., temperature, heart rate) were unaffected by noise levels, leading the authors to conclude that certain handling aspects during exams were a more powerful stressor than noise levels. Again, the differences between Stellato et al.'s (38) findings and the present study may be related to setting (veterinary clinic vs. home), but also to the nature of the sound stimuli to which dogs were exposed.

The survey results revealed a mismatch between the owners' perception of fearfulness in their dogs, and the amount of fearful behavior exhibited by their dogs, suggesting that many owners are underestimating fearfulness and anxiety in their dogs. While (as noted above) publicly-shared videos and compilations represent a biased sample, these videos demonstrate that some proportion of dog owners react to their dogs' fearful and defensive reactions with amusement, rather than concern. Concern was only expressed by owners/narrators in 17.5% of the individual videos, and was not expressed in any of the video clip compilations used in this study. Our Hypothesis 2 was thus also supported by these data: owners can misinterpret or respond negatively to expressions of fear, anxiety, or stress in their companion dog, particularly if the stressor is considered “common.” These findings underscore calls for better education for dog owners with regard to accurately interpreting canine body language, particularly the more subtle signs of stress and anxiety, to both safeguard welfare and minimize development of anxiety-related behavior problems [e.g., (39)].

Other studies have demonstrated that non-experts (i.e., the average companion dog owner, as opposed to a canine behaviorist or knowledge-assessed trainer) may be less able to identify signs of fear and anxiety in their dogs, particularly when the behaviors are subtle (39, 40). Because of this inability to accurately read canine body language, many owners appear to perceive their dogs as less fearful than they actually are, and may therefore not intervene in response to early or subtle stress signals to mitigate or remove sources of stress for their dogs (39). A particular extreme example of a dog's reaction to household noise bears mentioning here: in Bender and Strong (22), canine behavioral consultant Emily Strong describes a case working with a dog displaying signs of intense, chronic anxiety (pacing, whining, restlessness, lack of interest in interacting with his owners). The owners of this dog were mystified by the dog's behavior, and had spent thousands of dollars ruling out medical issues with this dog; no medical problems had been found. The behavior had started suddenly a few months prior to the first consult, and continued to get worse; the owners had lived with the dog since puppyhood, and prior to this change, the dog had never exhibited any anxiety-related or behavioral problems. Tellingly, the dog was relaxed and happy when away from the home, but resistant to returning home after walks. After much questioning, it was discovered that the owners had installed a sonic (very high frequency) pest repellant device in the home a few months before; upon turning off the device, the dog immediately began to relax, and returned to his normal behavior over the next few days. These owners were, by all accounts, very dedicated to the welfare of their dog, but they could not hear the device; the dog, on the other hand, was highly bothered by it [(22); p. 6]. Ultrasonic pest deterrents are generally inaudible to humans, although van Wieringen and Glorieux (41) noted that some younger individuals were able to hear them and found them disturbing, even during relatively short exposures (20 mins). Many everyday household devices produce ultrasound (42), and ultrasound, particularly when very loud, has been used successfully to repel dogs from tested areas (4). In humans, exposure to ultrasound has been associated with annoyance, disorientation, headache, fatigue, nausea and high arousal (31).

Individual dogs vary in their ability to cope with stressors (43), and not all dogs display marked sensitivity or phobias toward noise. Dogs suffering from physical pain may be at increased risk of noise sensitivity; Fagundes et al. (44) reported fear responses to loud noises in 10/10 noise-sensitive dogs concurrently experiencing pain, vs. in 6/10 noise-sensitive control dogs not experiencing pain. Up to 50% of dogs may experience noise sensitivity and/or extreme reactions to noise in their lifetimes (5, 7). Many companion dogs are primarily contained within human households (alone, or with human caretakers unable to detect these sounds, or to accurately interpret the dog's fearfulness), and are unable in many cases to control their own level of exposure to these sounds. This issue, therefore, may represent a significant welfare concern for affected dogs. In conclusion, and as noted earlier, additional targeted research into companion dogs' reactions to household sounds, owner perceptions of (and reactions to) their dogs' behavior, and the associated welfare implications, is warranted.

## Data Availability Statement

The raw data supporting the conclusions of this article will be made available by the authors, without undue reservation.

## Ethics Statement

The studies involving human participants were reviewed and approved by UC Davis Institutional Research Board. The survey participants provided their written informed consent to participate in this study.

## Author Contributions

LH: conceived the study. LH, EP, and AG-D: designed the initial approach and survey. EG: developed the ethogram and method of video analyses, conducted statistical analyses, and drafted initial manuscript. JC and SC: compiled data from videotapes. EG and LH: edited manuscript. All authors contributed to the article and approved the submitted version.

## Funding

This work was supported by the Center for Companion Animal Health, University of California, Davis (# 2009-54-F/M).

## Conflict of Interest

The authors declare that the research was conducted in the absence of any commercial or financial relationships that could be construed as a potential conflict of interest.

## Publisher's Note

All claims expressed in this article are solely those of the authors and do not necessarily represent those of their affiliated organizations, or those of the publisher, the editors and the reviewers. Any product that may be evaluated in this article, or claim that may be made by its manufacturer, is not guaranteed or endorsed by the publisher.

## Supplementary Material

Supplementary Material 1 23-Item survey (IRB # 1469462-1).

Supplementary Material 2 List of URLs of videos and compilations used in this study.
