# Supplementary material for: Stress-Related Behaviors in Companion Dogs Exposed to Common Household Noises, and Owners' Interpretations of Their Dogs' Behaviors
Source: Front Vet Sci. 2021 Nov 8;8:760845. doi: 10.3389/fvets.2021.760845 (PMC8606548; doi:10.3389/fvets.2021.760845)
Supplement: Supplementary Material 1 — 23-Item survey (IRB # 1469462-1). [file Data_Sheet_1.PDF]

UCDAVIS
UNIVERSITY OF CALIFORNIA

Exclusionary Questions

Do you currently own a dog?

- ○ Yes
- ○ No

Are you able to read and write in English?

- ○ Yes
- ○ No

Informed Consent

Welcome to the UC Davis Hart Lab!

We are conducting research to understand how dogs respond to different sounds. Our focus is to understand the behavior of pets at home, and how humans can become better owners. In this survey, we will ask a series of questions pertaining to your pet's history, behaviors, and any past therapies. Please be assured that your responses will be kept completely confidential and anonymous.

The study should take you around 5-10 minutes to complete. Your participation in this research is voluntary. You have the right to withdraw at any point during the study, for any reason, and without any prejudice. If you would like to contact the research team discuss this research, please e-mail Lynette Hart, lahart@ucdavis.edu   or phone 530-752-2181

By clicking the button below, you acknowledge that your participation in the study is voluntary, you are 18 years of age, and that you are aware that you may choose to terminate your participation in the study at any time and for any reason.

Pet Information

The name of your pet you would like to input information on for this survey (Note: if you have multiple pets in your household, please only submit information for your pet that most experiences issues with behavior or noises)

Age of pet

- ○ Less than 1 year
- ○ 1-2 years
- ○ 2-7 years
- ○ 7+ years

Breed of pet

Please select which weight range your pet falls into.

- ◯ <20 pounds
- ◯ 20-40 pounds
- ◯ 40-60 pounds
- ◯ 60-80 pounds
- ◯ 80+ pounds

Origin of pet

- ◯ Family/Friend
- ◯ Shelter/Rescue
- ◯ Breeder
- ◯ At home breeding
- ◯ [ ] Other

If you selected Shelter/Rescue above, do you know if your pet experienced fostering before adoption?

- ◯ Yes, I know my pet had been fostered prior to adoption
- ◯ No, I know my pet had not been fostered prior to adoption
- ◯ I do not know
- ◯ Not applicable

Do you have any other dogs in the household?

- ◯ Yes
- ◯ No

How long have you had your pet?

- ◯ Less than 1 year
- ◯ 1-2 years
- ◯ 2-7 years
- ◯ 7+ years

Has your pet even been diagnosed with deafness or any level of hearing impairment?

- ◯ Yes
- ◯ No

Has your pet ever been diagnosed with a serious or chronic condition that may affect your pet's daily comfort?

- ◯ No
- ◯ [ ] Yes

Pet Behaviors

Do you consider your pet to be anxious?

- ◯ Yes
- ◯ No
- ◯ I am not sure

Do you consider your pet to be fearful?

- ◯ Yes
- ◯ No
- ◯ I am not sure

Please check each box that indicates a behavior you see in your animal that you would consider common for your pet.

- ☐ Panting
- ☐ Yawning
- ☐ Barking
- ☐ Howling
- ☐ Whining
- ☐ Lip licking
- ☐ Salivation
- ☐ Pacing
- ☐ Tucked ears
- ☐ Tucked tail
- ☐ Shaking
- ☐ Retreating
- ☐ Hiding

Pet Behavior Specific to Sound

Please check each box that indicates a behavior you see in your pet when sounds occur in the home.

- [ ] Panting
- [ ] Yawning
- [ ] Barking
- [ ] Howling
- [ ] Whining
- [ ] Lip Licking
- [ ] Salivation
- [ ] Pacing
- [ ] Tucked Ears
- [ ] Shaking
- [ ] Retreating
- [ ] Hiding

Do you consider your pet to have a fear of loud noises? e.g., fireworks, thunderstorms, gunshots

- ○ Yes
- ○ No
- ○ I am not sure

What category of sounds do you feel your pet reacts to?

- ☐ Loud, rare sounds, e.g., fireworks, thunderstorms, gunshots
- ☐ Beeping sounds, e.g., alarm blocks, smoke detectors, cell phone noises
- ☐ Appliance sounds, e.g., washing machine/dryer/dishwasher, vacuum, fans, plumbing
- ☐ __________ Other:

Please describe, pertaining to your answer above, the specific sounds that evoke stress, fear, or anxiety in your animal.

____

Actions

Has your dog ever participated in training classes / group training sessions?

- ◯ Yes
- ◯ No

Have you ever taken your pet to a veterinarian or an animal behaviorist for behavior therapy for a specific issue?

- ◯ Yes
- ◯ No

Have you ever treated your pet for anxiety?

- ◯ Yes
- ◯ No

If you have used any of the following anti-anxiety therapies, please rate the success level of those treatments.

	Have not tried	Significantly worsened anxiety	Somewhat worsened anxiety	No change	Somewhat successful in reducing anxiety	Significantly successful in reducing anxiety
Medication	○	○	○	○	○	○
Pheromone / Aroma Therapy	○	○	○	○	○	○
Supplements	○	○	○	○	○	○
Nutrition changes	○	○	○	○	○	○
Massage / Touch Therapy	○	○	○	○	○	○
Acupuncture	○	○	○	○	○	○
Thundershirt	○	○	○	○	○	○

If you have used any of the therapies above, please indicate where you were introduced to the therapy.

- ☐ Veterinarian
- ☐ Behaviorist/Specialist
- ☐ Online
- ☐ Family/Friend
- ☐ Pet Store
- ☐ Book
- ☐ Television

Household Noises

Has your pet ever experienced an extreme response to a noise from within your home? If yes, please describe in detail.

Powered by Qualtrics
