# Supplementary material for: Stress-Related Behaviors in Companion Dogs Exposed to Common Household Noises, and Owners' Interpretations of Their Dogs' Behaviors
Source: Front Vet Sci. 2021 Nov 8;8:760845. doi: 10.3389/fvets.2021.760845 (PMC8606548; doi:10.3389/fvets.2021.760845)
Supplement: Supplementary Material 2 — List of URLs of videos and compilations used in this study. [file Data_Sheet_2.PDF]

Stress-Related behaviors in companion dogs exposed to common household noises, and owners' interpretations of their dogs' behaviors.
Supplementary Material (B)
VIDEO CLIPS:

Video#	Link	Original Posting Date	Video Title
1	https://www.youtube.com/watch?v=9rJuz7pnTr0	8-Dec-13	Dogs reaction to smoke alarm
2	https://youtu.be/VFrfgmhcrc0	23-Nov-12	Funny dog reaction to smoke alarm.
3	https://www.youtube.com/watch?v=lJ8MPlxsVIA	28-Jun-13	Dog Reacting to Smoke Detector Chirp
4	https://www.youtube.com/watch?v=pLkHNBjX1r0	18-Apr-17	My dog reacting to smoke alarm (WARNING LOUD NOISE)
5	https://www.youtube.com/watch?v=sm4hRg5UAdI	12-Oct-15	Dogs hear smoke detector.
6	https://www.youtube.com/watch?v=pNlMmulewjA	21-Nov-11	Dogs reaction to fire alarm
7	https://www.youtube.com/watch?v=ziofoWfGicw	1-Nov-09	Sasha scared of the smoke alarm!
8	https://www.youtube.com/watch?v=THKMIZl90UY	23-Jan-18	German Shepherd Howls During Fire Alarm | Pets Reacting to Alarm
9	https://www.youtube.com/watch?v=zlF-ERoBGD0	16-Oct-14	Dog howls at smoke alarm
10	https://www.youtube.com/watch?v=M5vc6Lec7kU	26-Jul-12	Dog howling at smoke alarm.
11	https://www.youtube.com/watch?v=OyXTJJtABw0	18-Aug-17	Nacho scared of beeping smoke alarm
12	https://www.youtube.com/watch?v=h0Tvk7vcmog	17-Sep-17	My dog going crazy with the smoke detectors
13	https://www.youtube.com/watch?v=20ALpPtYHmc	11-Jun-13	Chadrin chasing smoke alarm chirp
14	https://www.youtube.com/watch?v=xnVQ9gJrYFY	Mar 27, 2013	dogs reaction to fire alarm
15	https://www.youtube.com/watch?v=u80fenpUKdU	Oct 10, 2009	Insane Dogs Response to a Smoke Detector
16	https://www.youtube.com/watch?v=cgLclvOIKa8	May 12, 2012	Dog barking at Smoke Detector
17	https://www.youtube.com/watch?v=c3a3PfY0soA	May 18, 2015	Dogs vs.a stupid smoke alarm
18	https://www.youtube.com/watch?v=4wDhTfQYu5c	Dec 26, 2012	dog goes crazy over smoke alarm
19	https://www.youtube.com/watch?v=gnXJVpkq5j4	Apr 3, 2014	Smoke alarm beep
20	https://www.youtube.com/watch?v=gz3bzXzr8I4	May 24, 2012	Smoke alarm dog
21	https://youtu.be/TqOukjw5U-c	Jun 12, 2018	MY dog hates the sound of my microwave
22	https://youtu.be/TdoVSxx_eDY	Aug 16, 2017	Dog scared of microwave
23	https://youtu.be/V7HwCv9AzSY	Jul 30, 2011	Golden Retriever is afraid of the microwave
24	https://youtu.be/6t244575Dso	Feb 21, 2012	Dog scared of popcorn
25	https://youtu.be/yFmG_TpqH2w	Mar 27, 2011	Dog singing to smoke detector
26	https://youtu.be/ZUKmSiytu2o	Jun 7, 2013	Scared of the Smoke Alarm
27	https://youtu.be/WKUG1VVOc10	Feb 25, 2013	"Jack" the Dog hates smoke alarms
28	https://youtu.be/K5Ee8ru9-6g	Feb 3, 2014	My dog likes to howl when the smoke alarm goes off.
29	https://youtu.be/iJr5t4EfHD0	Dec 11, 2012	Dog howling at smoke alarm
30	https://youtu.be/4uRWv33bbQc	May 31, 2015	Smoke Detector Chirp Chickens
31	https://youtu.be/g6iPrAe0Pc0	Sep 15, 2008	brany(my dog) barking at the smoke alarm
32	https://www.youtube.com/watch?v=EMrCc0bfwes	Mar 26, 2014	dog hates the beep sound
33	https://youtu.be/aNSSkcNYrk4	Oct 29, 2013	Zani in Extreme Fear
34	https://www.youtube.com/watch?v=mFvc475QBAE	Aug 9, 2016	Dog goes crazy over vacuum noise
35	https://www.youtube.com/watch?v=xh2kzi6BU9M	6-May-13	Dog reacts to fire alarm
36	https://www.youtube.com/watch?v=tgEBp-6vPqo	8-Nov-19	(ENG Sub) Dog Reaction to Fire Alarm
37	https://www.youtube.com/watch?v=FIP0Qc7AMo8	6-Jul-12	How Dogs React to a fire alarm
38	https://www.youtube.com/watch?v=20mbXYFWwgw	30-Jun-11	My pug howling with the fire alarm in San Francisco
39	https://www.youtube.com/watch?v=BRDPPtgR9hs	10-Dec-19	My dog reacting to smoke detector
40	https://www.youtube.com/watch?v=e9jznolJx2U	24-Oct-13	MY DOG CRYING, SCARED OF THE FIRE DETECTOR lol
41	https://www.youtube.com/watch?v=_ufB7F4t19c	1-Jan-11	dogs listening to smoke detector
42	https://www.youtube.com/watch?v=D2NNrCYYm_o	3-Dec-15	Dog is not a fan of fire alarms
43	https://www.youtube.com/watch?v=x9qEDENAYpM	31-Mar-10	Crazy Dog hears the fire alarm
44	https://www.youtube.com/watch?v=rnsTCSpAkCs	12-Apr-13	Dogs Howling at Fire detector
45	https://www.youtube.com/watch?v=gPRmedVtbKM	28-Feb-12	My Dog cryin cuz da smoke detector went off
46	https://www.youtube.com/watch?v=3oc1gbnXTlQ	30-Oct-12	Azul hates smoke detector
47	https://www.youtube.com/watch?v=GcR6A6EK3SQ	22-Jan-11	Hilarious moment Dog howling at the fire alarm tests
48	https://www.youtube.com/watch?v=BXjkRXMbR9w	26-Feb-12	Monty barking At cmoke detector
49	https://www.youtube.com/watch?v=fwKmmL08mMg	12-Jun-09	Let your dog sing along…Muppet sings with the smoke alarm
50	https://www.youtube.com/watch?v=RYUFzQh-nXg	11-Feb-15	Rex reaction to smoke detector
51	https://www.youtube.com/watch?v=WsvpM36qBz4	30-Oct-19	Hidden Smoke Alarm Gace my dog Crazy Anxiety!
52	https://www.youtube.com/watch?v=0wcafMfPZDg	26-Aug-13	Mitzi howling at smoke detector
53	https://www.youtube.com/watch?v=h6k2Rr-O-Cs	7-Oct-16	Dog howling to fire alarm
54	https://www.youtube.com/watch?v=NEyUd3EzvoY	14-Aug-18	My Dogs Barking At the Fire Alarm
55	https://www.youtube.com/watch?v=j9HXD6C5bkg	20-Jun-12	Toto the dog getting scared of the fire detector
56	https://www.youtube.com/watch?v=y9RC4VAhEMo	27-Feb-09	Sadie and the smoke detector
57	https://www.youtube.com/watch?v=FPlM_0Y89S4	16-Feb-09	Dog vs. Roomba
VIDEO COMPILATIONS:			
C1	https://www.youtube.com/watch?v=PjQoyC9fhi8&ab_channel=FunnyPets	6-Nov-18	Dogs Vs Vacuum 😂🐶 Funny Dog's Reactions to Vacuum (Full) [Funny Pets]
C2	https://www.youtube.com/watch?v=mLBF04hj8Uw&t=12s&ab_channel=MrFunnyMals	9-Dec-16	Dogs vs. Roombas
C3	https://www.youtube.com/watch?v=r4YMsjkeR1s&ab_channel=Rufus	11-Apr-20	Roomba Animals! Funny Videos of Pets and Roombas
C4	https://www.youtube.com/watch?v=cPgYKSuRihE&ab_channel=PETacular	13-Nov-18	Funny Dogs Afrain Of Vacuums - HILARIOUS Animal Video Compilation [NEW HD]
C5	https://www.youtube.com/watch?v=8gEbQEwSZHQ&t=40s&ab_channel=Newsflare	7-Apr-19	Dogs vs Vacuum Cleaners Compilation | The Ultimate Showdown
